# Effects of a physical activity intervention on schoolchildren fitness

**DOI:** 10.14814/phy2.15115

**Published:** 2022-01-25

**Authors:** Antonio Di Maglie, Santo Marsigliante, Giulia My, Salvatore Colazzo, Antonella Muscella

**Affiliations:** ^1^ Department of History, Society and Human Studies University of Salento Lecce Italy; ^2^ Department of Biological and Environmental Science and Technologies (DiSTeBA) University of Salento Lecce Italy

**Keywords:** BMI, children obesity, overweight, physical activity, waist circumference

## Abstract

The global prevalence of childhood obesity is high. Obesity main causes are linked to sedentary lifestyles. Increasing physical activity (PA) and reducing sedentary activities are recommended to prevent and treat obesity. This study aimed to evaluate the effectiveness of a 6‐month school PA intervention on obesity prevention and healthy behaviors in school‐aged children. Participating students (10–11 years of age) were randomly divided into an intervention group and a control group. Children in the intervention group (*n* = 80) participated in a multicomponent PA that included improvement in extracurricular physical activities (with an additional 40 min per day for 5/6 days per week). Children (*n* = 80) in the control group participated in usual practice. Participants had mean body mass index of 19.7 ± 2.9 kg/m^2^, and 33.7% of them were overweight or with obesity at T0. The change in body mass index in intervention group (−2.4 ± 0.6 kg/m^2^) was significantly different from that in control group (3.01 ± 1.8 kg/m^2^). The effects on waist circumference, waist‐to‐height ratio, and physic fitness were also significant in intervention group compared with control group (all *p* < 0.05). Furthermore, there is a significant decrease in overweight or children with obesity in the experimental group (to 17.5%, *p* < 0.05). These findings suggest that a school‐based intervention program represents an effective strategy for decreasing the number of overweight and children with obesity.

## Graphical abstract

Lifestyle changes toward an increased physical activity (PA) and reduced sedentary activities are recommended to prevent and treat obesity. After 6‐month of increased PA the children included in participant group significantly improved body mass index, waist circumference, waist‐to‐height ratio, and physic fitness; instead, no such improvements were observed for the nonparticipant group. Our data suggest that a school‐based intervention program represents an effective strategy to reduce overweight and obesity in children.

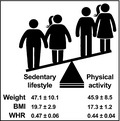

## 1 INTRODUCTION

Recently, the high prevalence of obesity among children and adults is an important public health issue (Ingle et al., 2006). In fact, obesity, low level of physical activity (PA) together with dysfunctional movement in childhood, can contribute to even more increased risk for developing not only metabolic and cardiovascular disease, but also musculoskeletal health in adulthood (Hall et al., 2018; Karuc & Mišigoj‐Duraković, 2019). Several modifiable behaviors have been found to influence the development of obesity in children; generally, insufficient PA and high caloric intake are considered important contributors to the recent increases in obesity among youth (Ingle et al., 2006; Minck et al., 2000; Van der Heijden et al., 2010). Furthermore, a negative impact of higher weight status on motor coordination performance, according to age and gender, has also been shown (Battaglia et al., 2021). In Italy, the most likely causes for the development of obesity include an increased consumption of fats, particularly saturated fats, sugars, a reduced intake of fiber, as well as a progressive sedentary behavior (Minck et al., 2000). It is important to note that trends over the past few decades demonstrate decreased health‐related fitness levels in youth across the world (Lopes et al., 2012; Rodrigues et al., 2013, 2016). With social development and changes in people's lifestyles, children and adolescents currently do not practice adequate PA (Dobbins et al., 2009; Sallis et al., 2000). In addition, parents may themselves be overweight or with obesity, suggesting that family environments may be obesogenic in nature (Bülbül, 2020). Health‐promoting interventions aimed at improving PA through exercise programs have shown promising results in different life stages (Yuksel et al., 2020). The intervention during childhood, when negative weight‐related behaviors are acquired, can result advantageous in preventing obesity (Kim et al., 2020; Sung et al., 2019). Welfare of children in Italy was promoted by the Italian Ministry of Health's Physical Activity Guidelines since for children and young people PA is essential for healthy bones and joints, muscular system development, the control of body weight and the proper functioning of the cardiovascular, and respiratory systems (Musumeci, 2016). Unfortunately, children are frequently reluctant to comply with the exhortations aimed at controlling their body weight. Since children spend a great deal of time in school, the implementation of school‐based programs in promoting PA could be important to establish and maintain healthy lifestyle (Von Hippel et al., 2007; Ip et al., 2017) and to increase the cognitive functions and the mental health (Claver et al., 2017). The WHO specifically identified schools as a target setting for the promotion of PA among children and youth (Dobbins et al., 2009). While some studies targeting school PA (Van Barbeau et al., 2007; Kriemler et al., 2010; de Kop et al., 2019) have shown positive effects, others have given disappointing results (Harris et al., 2009; Hung et al., 2015; Hynynen et al., 2016). Although the nutritional component was adopted in some interventions, the results did not show a greater effect of PA plus nutrition than PA alone (Lavelle et al., 2012; Waters et al., 2011). In addition, in most school‐based intervention programs (Jiang et al., 2007; Li et al., 2014; Liu et al., 2007), PA component was adopted as main strategy, while the nutrition component mostly acted as subsidiary part.

The aim of this study was to develop a childhood obesity prevention intervention in two schools in Southern Italy and to examine the 6‐month effect of this pilot intervention on students’ anthropometry, body composition, and fitness. As part of the intervention, in addition to normal sports activities, we have enriched the PA program. Due to the lack of canteens in these schools, which makes it not feasible to adopt a nutritional intervention and calculate nutritional intake in the school environment, we have not included the nutritional component in our intervention. Instead, we included some components of healthy eating in health education lectures.

## 2 METHODS

### 2.1 Participants

To understand the effects of promoting PA intervention, we selected a sample of 160 children, aged 11.5 ± 0.5 years, belonging to two schools. These schools have never participated in health promotion programs and are located in two cities with similar socio‐economic status. Schools were secondary level public schools, with attendance from 8:00 to 13:00.

**FIGURE 1:**
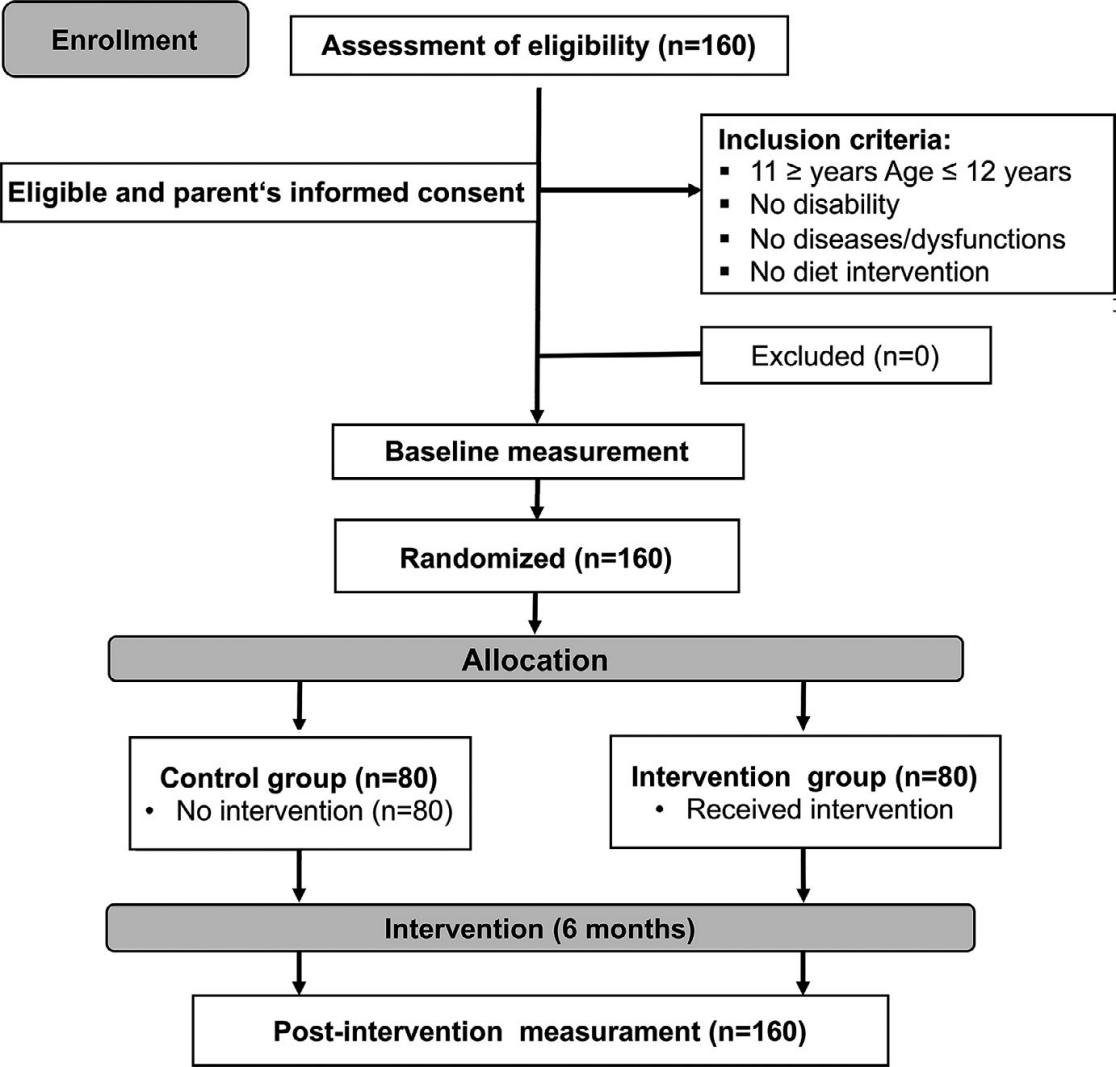
Scheme of the participants in the study

The sample was composed of 80 boys and 80 girls (aged 11.4 ± 0.5, and 11.5 ± 0.5, respectively) who were assigned to a control group and an intervention group (*n* = 80 and *n* = 80, respectively). All children were healthy and free of any disability or musculoskeletal, cardiological, neurological or respiratory diseases, or dysfunctions (Figure 1). Before the inclusion of the children in our program, a parent or legal representative of each child signed an informed consent. The study was conducted in accordance with the Helsinki Declaration and the European Union recommendations for Good Clinical Practice (document 111/3976/88, July 1990). The University's Research Ethics Committee approved the study.

### 2.2 Intervention program

A physical education teacher was responsible for implementing the PA program and evaluating the process. The teacher was also responsible for collecting anthropometric data during the 6‐month intervention period. Prior to the start of the project, all intervention school teachers received on‐site training to provide them with general information on the nature and significance of the intervention. Directly involved teachers and parents received training from the physical education teacher to support their role in the education of children. The procedures and training methods were standardized and shared by each school. The training, lasting 8 h and delivered over 2 days, included a theoretical and a practical part. The program lasted six consecutive months in schools, sports centers, and their own homes.

Participants in this study were children regularly practicing school physical education and/or sporting activities such as basketball, soccer, swimming, and volleyball. Therefore, the intervention group added the following procedure to the practiced PA, while the control group did not. The enriched activity was obtained by limiting the inactivity time of children by introducing additional minutes of PA per day (at least 40 min) for 5\6 days a week for 6 months, in the context of schools and a sport center.

The intervention program with exercises is shown in Table 1. The children were continually stimulated to perform the PA intervention and the compliance of program was tracked by teacher and parents. Children in the control group participated in usual practice.

**TABLE 1:** The intervention program

Type of exercise	Time (min)
Warm‐up exercises	
Slow running	3
Physical assessment test preparation	
Standing high jump	7
Standing long jump	
Rope jump	
Training exercises	
Endurance running (50 m × 8 shuttle run)	15
30 m sprint	
Push‐ups	
Push‐ups on the legs	
Abdominals	
Preparation to sport	
Side run, back run, forward run, split run	10
Run with change of direction	
Hand‐joined ball throw	
Right‐hand and left‐hand throwing	
Receiving the ball	
Hops with feet together	
Hops with right and left foot	
Stretching	
Upper limbs	5
Lower limbs	
Spine	

### 2.3 Measures

All measures were recorded at baseline (T0) and at 6 months from baseline (T1, at the completion of the intervention).

The children's height was measured, in duplicate, using a portable stadiometer (SECA 213; Intermed). Body weight was ascertained, in duplicate, with standard techniques (Seca 700; Intermed). Waist circumference was measured, in duplicate, at the iliac crest, at mid‐respiration using a nonelastic measuring tape (Gulick II; Country Technology Inc.). The ratio between waist circumference and height (WHR) was calculated. The cut‐off used to represent CV risk for WHR was 0.500 (McCarthy & Ashwell, 2006). For children and adolescents, the Center for Disease Control and Prevention defines overweight as a body mass index (BMI: weight in kilograms divided by height in meters squared) between the 85th and 95th percentiles and obesity as a BMI at or above the 95th percentile for sex and age (Kuczmarski et al., 2002).

Parents completed a questionnaire developed by the authors, that dealt with children's daily frequency of physical education, number of hours of television viewing, hours of time spent on the computer, and hours spent playing electronic games. To get information regarding the PA improvements, the participants completed pre‐ (T0) and post‐intervention (T1) tests that have been frequently used in similar studies. Each test assesses different components of physical fitness (Stodden et al., 2015; Tabacchi et al., 2016) the Sargent Jump Test (Sargent, 1921), also known as the vertical jump test, to monitor the development of the athlete's elastic leg strength. To assess lower limb explosive power, we used the standing broad jump test (SBJ) (Davies, 1990). With number of Jump rope test for 1 min each student test their endurance (Buchheit et al., 2014). The schoolchildren performed each test three times, of which the best result was considered in the analysis. The children conducted the tests in a random order. After T0, the children were randomly assigned to the control group or intervention group, respectively. After the interventions period, (T1) the participants were re‐evaluated, at a similar time of T0.

### 2.4 Statistical analysis

Means and standard deviations were described for the total sample and for both the intervention and control groups. All data have been tested for normality using the Shapiro–Wilk test. Since the data appears to be not normally distributed, nonparametric evaluation has been carried out. To identify differences between groups, the Mann–Whitney test was performed. The paired sample Student's *t*‐test was used to test differences in anthropometric data before and after intervention. Analysis of the qualitative associations between parameters was performed using the Fisher's exact test. Significance level was set to *p* < 0.05.

## 3 RESULTS

### 3.1 Characteristics of the participants

The baseline sample included 160 children from both intervention and control groups (Figure 1). No children dropped out after randomization. Baseline, follow‐up values for anthropometry and fitness variables were collected on total population as detailed under Methods. Table 2 shows the baseline characteristics of 80 children in the intervention group and 80 in the comparison group. The average age was 11.5 ± 0.5 years for both sexes. Consistently with a random assignment of schools to experimental and control groups, no significant differences between groups were found in mean values for weight, height, BMI, and proportion of overweight or children with obesity (*p *> 0.05 by Mann–Whitney test; Table 2). No statistically significant differences between the sexes were noted (*p *> 0.05 by Mann–Whitney test).

**TABLE 2:** Physical characteristics of children

	All (*n* = 80)	Boys (*n* = 39)	Girls (*n* = 41)
Characteristic (participant group)			
Age, years	12.1 ± 0.5	12.6 ± 0.5	11.8 ± 0.5
Height, cm	154.2 ± 7.6	154.9 ± 9.4	153.4 ± 6.2
Weight, kg	47.1 ± 10.1	48.1 ± 5.8	45.8 ± 9.6
BMI, kg/m^2^	19.7 ± 2.9	20.2 ± 1.2	19.1 ± 2.9
BMI, percentile	63.3 ± 24.2	65.5 ± 23.5	60.8 ± 25.6
WHR	0.47 ± 0.06	0.48 ± 0.06	0.46 ± 0.05
Characteristic (nonparticipant group)			
Age, years	11.49 ± 0.5	11.41 ± 0.5	11.6 ± 0.5
Height, cm	153.8 ± 8.1	153.9 ± 9.4	152.2 ± 6.8
Weight, kg	46.05 ± 7.9	46.56 ± 9.3	43.9 ± 5.9
BMI, kg/m^2^	19.2 ± 2.7	19.7 ± 2.9	18.5 ± 2.4
BMI, percentile	64.0 ± 23.8	65.1 ± 25.6	58.9 ± 25.9
WHR	0.46 ± 0.07	0.47 ± 0.04	0.45 ± 0.08

Note

Values are mean ± SD.

Abbreviations: BMI, body mass index (calculated as weight in kilograms divided by height in meters squared); WHR, waist‐to‐height ratio.

**FIGURE 2:**
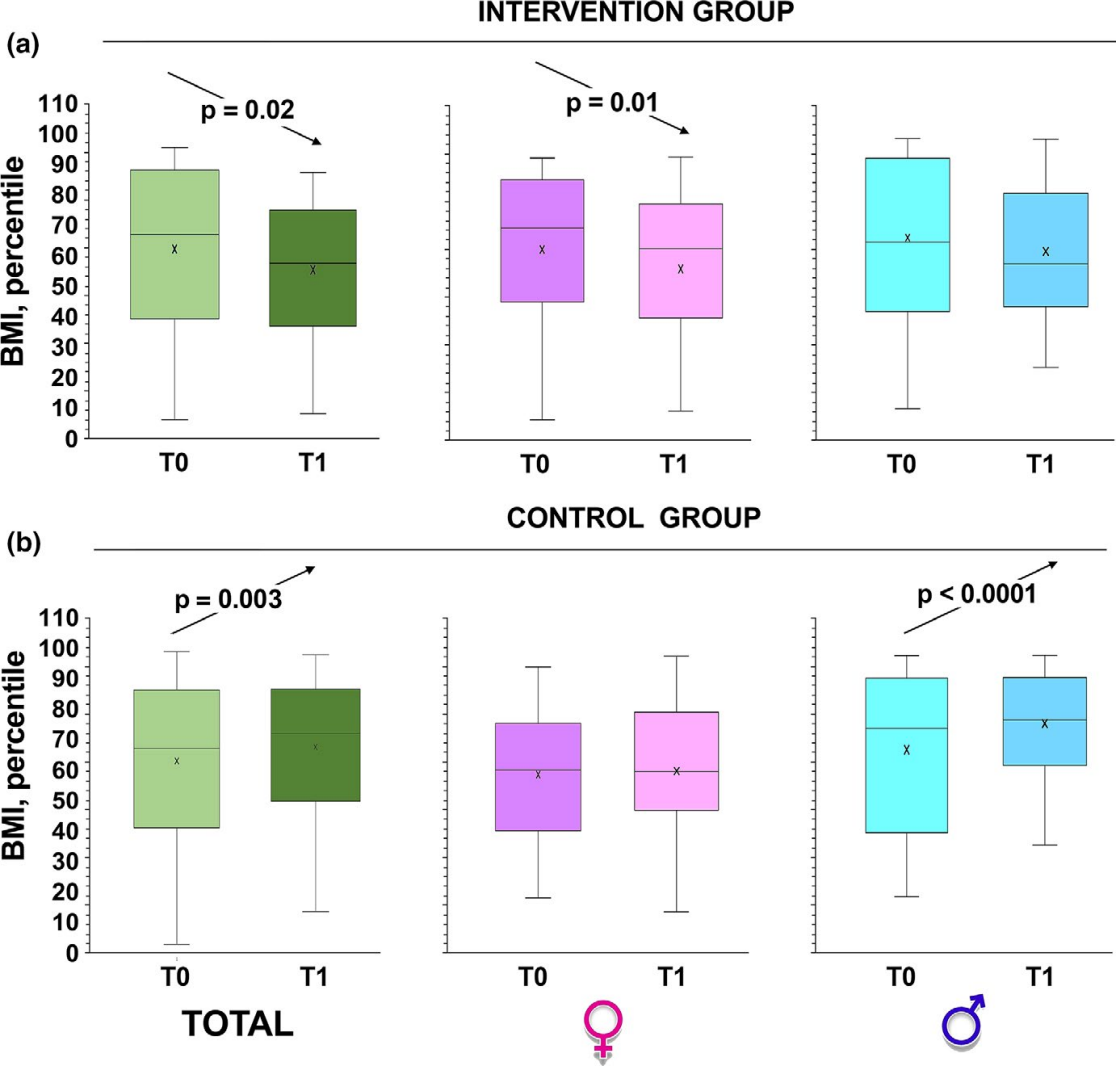
Changes in BMI percentile over time. Box and whiskers representation of body mass index (BMI: weight in kilograms divided by height in meters squared) of intervention group (a) and control children (b), recorded at baseline (T0) and at 6 months from baseline (T1, at the completion of the intervention). The sample was also divided according to gender. In this representation, the central box covers the middle 50% of the data values, between the upper and lower quartiles. The bars extend out to the extremes, while the central line is at the median. *p*‐values were obtained by Student's paired *t*‐test

Interestingly, while in the untreated group BMI percentile increased (*p* = 0.02 by paired Student's *t*‐test; Figure 2a) it significantly decreased in intervention group (*p* = 0.003 by paired Student's *t*‐test; Figure 2b). On the other hand, there were significant differences between proportion of overweight, underweight, normal weight, or children with obesity pre‐ and post‐intervention, in experimental group (*p *= 0.0001 by Fisher's exact test; Table 3). In fact, children with obesity decreased from 10 to 4 and at the same time the normal weight children increased from 51 to 66 (Table 3). The best results were observed in the female sex, since there were no girls with obesity after the intervention (Table 3).

**TABLE 3:** Number of overweight, underweight, normal weight, or with obesity children with pre‐(T0) and post‐(T1) intervention

	T0			T1		
	All	Boys	Girls	All	Boys	Girls
Characteristic (participant group)						
Underweight	2	0	2	0	0	0
Normal weight	51	25	26	66	29	37
Overweight	17	9	8	10	6	4
Obesity	10	5	5	4	4	0
Characteristic (nonparticipant group)						
Underweight	2	1	1	2	1	1
Normal weight	51	24	27	48	22	26
Overweight	18	9	9	19	10	9
Obesity	9	5	4	11	6	5

**FIGURE 3:**
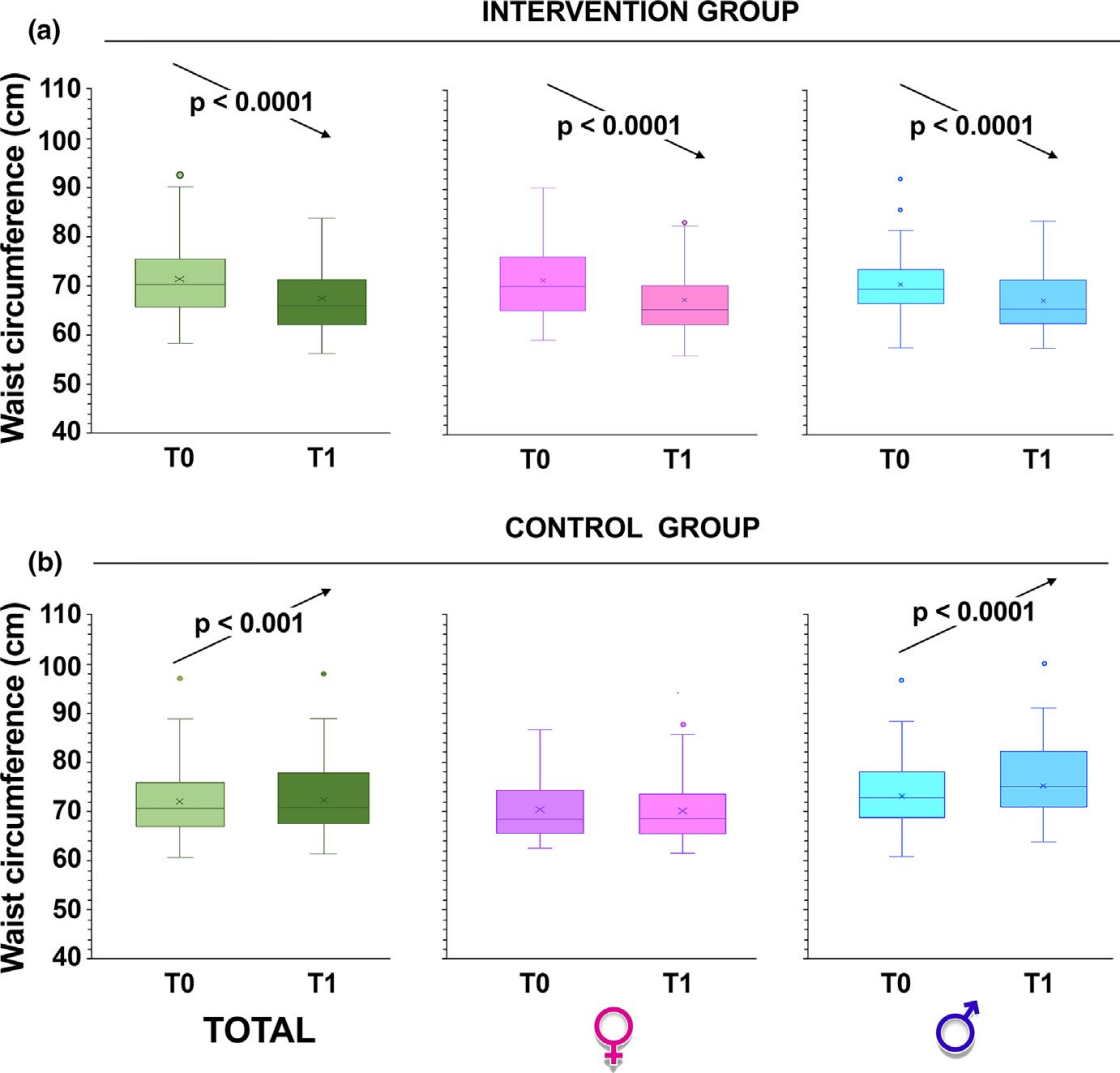
Changes in waist circumference over time. Box and whiskers representation of waist circumference of intervention group (a) and control children (b), recorded at baseline (T0) and at 6 months from baseline (T1, at the completion of the intervention). The sample was also divided according to gender. In this representation, the central box covers the middle 50% of the data values, between the upper and lower quartiles. The bars extend out to the extremes, while the central line is at the median. Those values which are beyond 1.5 times the interquartile range beyond the central box are plotted as individual points. *p*‐values were obtained by Student's paired *t*‐test

Waist circumference was also measured as another indicator of obesity; this is, in the experimental group, substantially decreased (*p* < 0.001 by paired Student's *t*‐test; Figure 3a). On the contrary, in the group not participating in the intervention, the waist circumference increased significantly, especially in boys (*p* < 0.0001 by paired Student's *t*‐test; Figure 3b).

WHR is believed to be an important indicator of excessive upper body fat, even in children of normal weight, and is therefore more sensitive than BMI in identifying children at risk of developing metabolic complications (McCarthy & Ashwell, 2006). A WHR cut‐off value was established as higher than >0.500 (McCarthy & Ashwell, 2006; Zhu et al., 2016) and, in T1, the mean WHR value decreased from 0.47 ± 0.06 to 0.43 ± 0.03 (*p* < 0.001 by paired Student's *t*‐test) in the intervention group, while it remained unchanged in the control group. In addition, the number of children who in T0 had a WHR >0.50 also significantly decreased (*p* < 0.0001 by Fisher's exact test; Table 4).

**TABLE 4:** Number (and percentage) of children with waist‐to‐height ratio, WHR >0.50, in pre‐(T0), and post‐(T1) intervention

Characteristic	T0			T1		
	All	Boys	Girls	All	Boys	Girls
Participant group						
WHR > 0.50	19 (24%)	11 (28%)	8 (19.5%)	6 (7.5%)	4 (10%)	2 (5%)
Nonparticipant group						
WHR > 0.50	18 (22.5%)	(10 26%)	8 (19.5%)	17 (21%)	9 (23%)	8 (19.5%)

### 3.2 Questionnaire results

Data on the children's weekly attendance of physical education, the number of hours of television viewing, hours spent on the computer and those spent playing electronic games are presented in Table 5.

**TABLE 5:** Descriptive statistics and *p* values for children's daily hours of time spent on PA, electronic games, television (TV), computer use pre‐(T0), and post‐(T1) intervention

Activities	No. children (T0) (%)	No. children (T1) (%)	*p*
Participant group			
Physical education/sport			
Yes	59 (73.8)	79 (98.8)	3.7 × 10^−7^
No	21 (26.3)	1 (1.2)	
Electronic games			
1–2 h	30 (37.5)	28 (35)	1.8 × 10^−11^
>2 h	20 (25)	5 (6.25)	
TV			
1–2 h	20 (25)	24 (30)	0.008
>2 h	16 (20)	6 (7.5)	
Computer use			
0 h	4 (5)	6 (7.5)	0.04
>1 h	62 (77.5)	48 (60)	
Nonparticipant group			
Physical education/sport			
Yes	56 (70)	59 (73.8)	0.46
No	24 (30)	21 (26.2)	
Electronic games			
1–2 h	30 (37.5)	29 (36.2)	0.47
>2 h	18 (22.5)	21 (26.2)	
TV			
1–2 h	20 (25)	21 (26.2)	0.46
>2 h	17 (21.2)	19 (23.7)	
Computer use			
0 h	5 (6.25)	5 (6.25)	0.80
>1 h	60 (75)	62 (77.5)	

As for the PA, a very large percentage (72.1%) practice physical education or sport; this percentage value increases considerably in the intervention group (from 73.8% to 98.8%). Notably, there was an increase in children who chose to move on foot or by bicycle and a decrease in those who moved with motorized vehicles.

As for sedentary behaviors, most children use computers for short periods and electronic games for longer periods.

As for TV, children spend more than 2 h a day watching it. In the experimental group, after our intervention, there was a significant decrease in the hours spent with video games/PCs/smartphones/tablets/TVs and at the same time an increase in the afternoon hours dedicated to sport (Table 5).

### 3.3 Physical fitness

**FIGURE 4:**
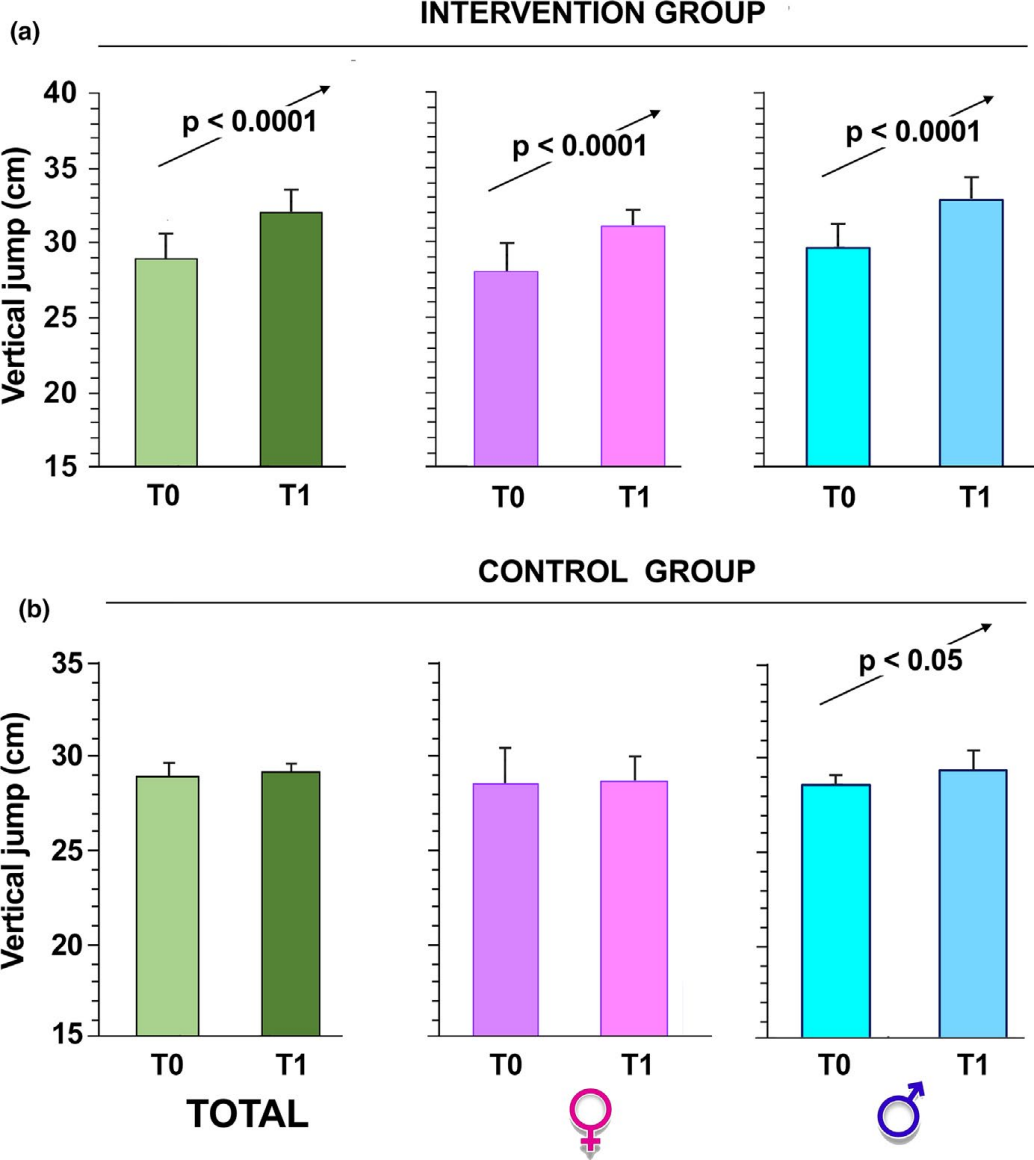
Vertical jump performances over time. The children, intervention group (a) and control group (b), were evaluated at T0 and 6 months after (T1, at the completion of the intervention). The sample was also divided according to gender. The data are presented as mean ± SD and significant differences between groups were evaluated by Student's paired *t*‐test

**FIGURE 5:**
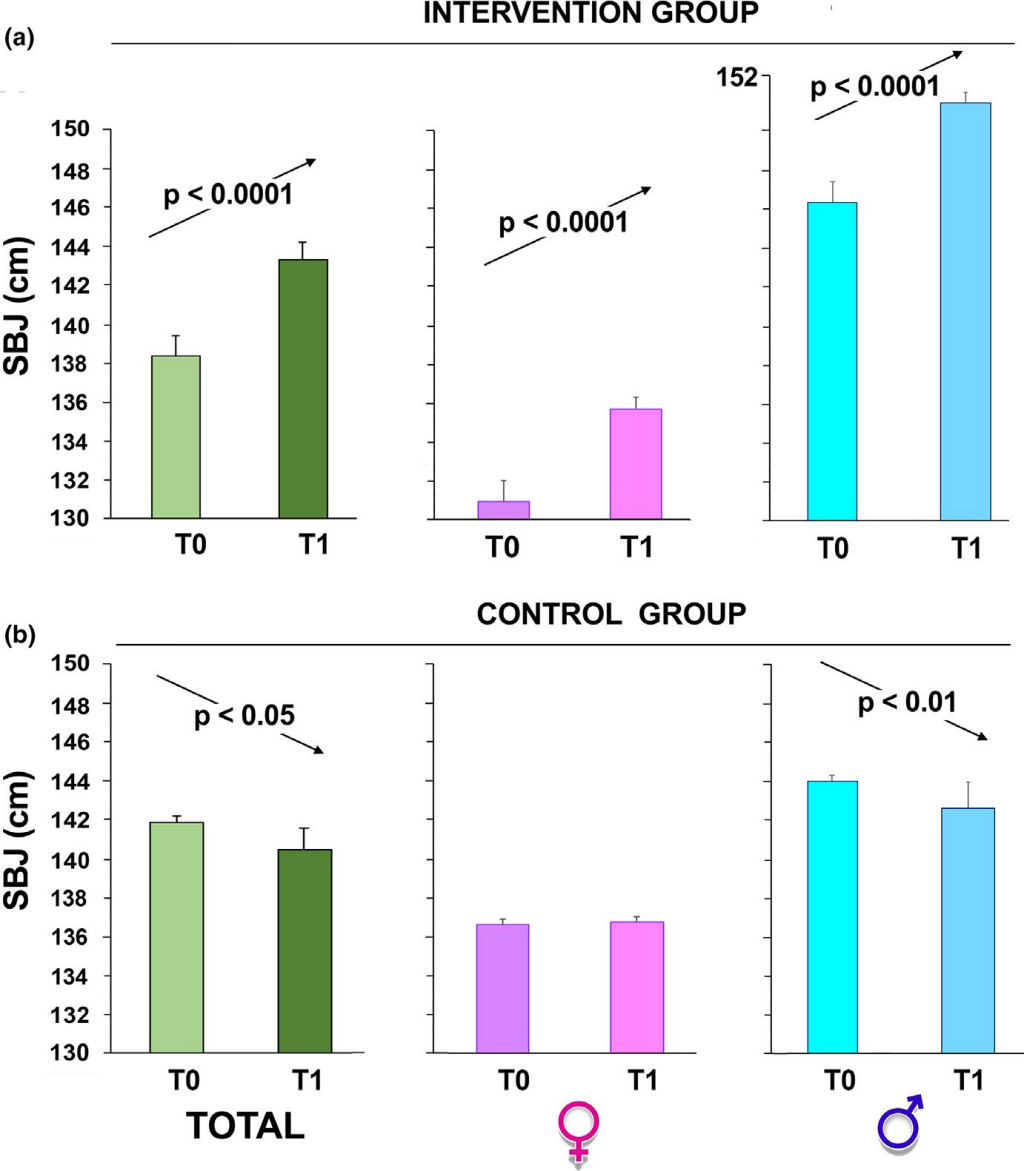
Standing broad jump test (SBJ) performance over time. The children, intervention group (a) and control group (b), were evaluated at T0 and 6 months after (T1, at the completion of the intervention). The sample was also divided according to gender. The data are presented as mean ± SD and significant differences between groups were evaluated by Student's paired *t*‐test

**FIGURE 6:**
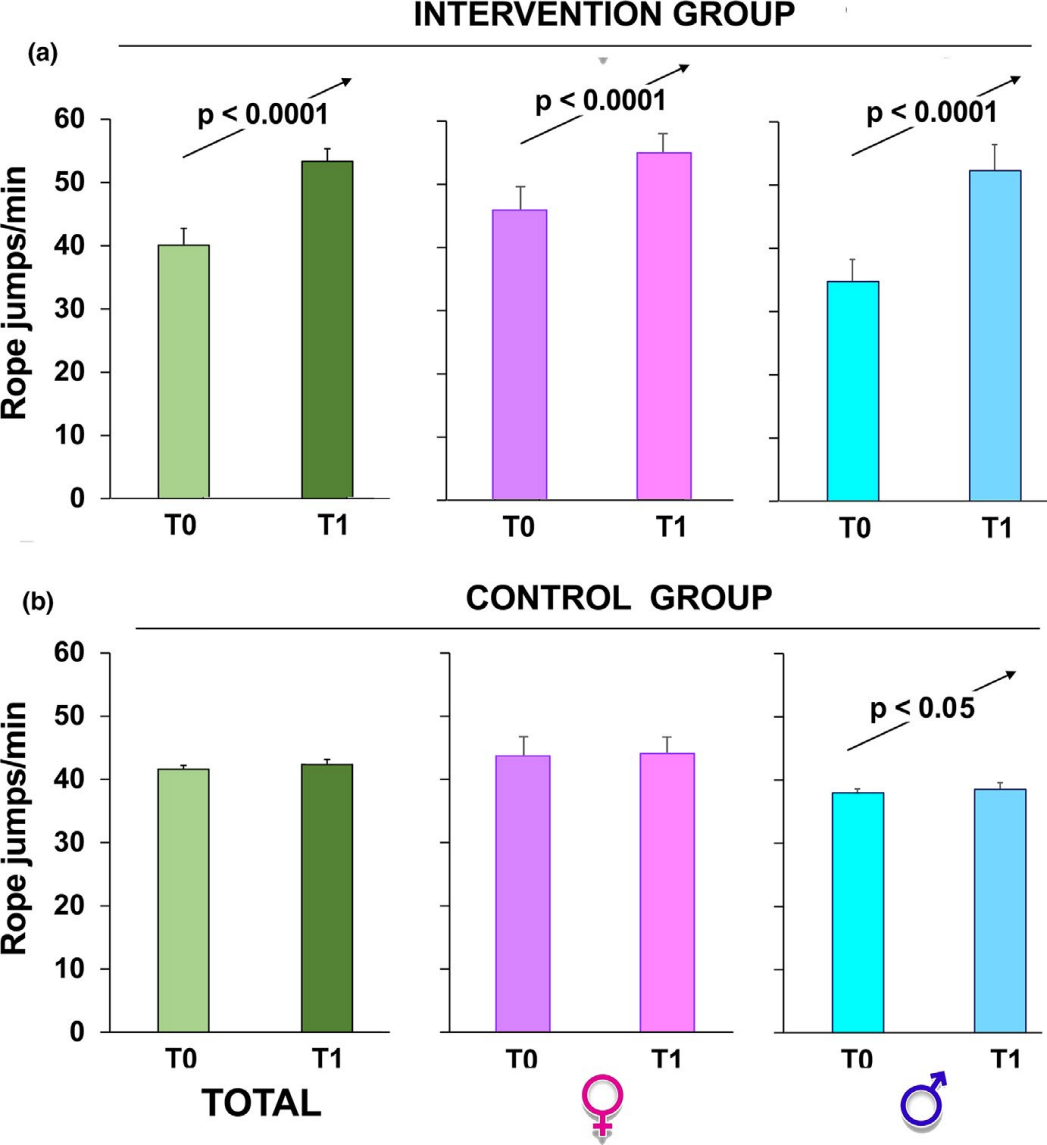
Rope jumps/min number over time. The children, intervention group (a) and control group (b), were evaluated at T0 and 6 months after (T1, at the completion of the intervention). The sample was also divided according to gender. The data are presented as mean ± SD and significant differences between groups were evaluated by Student's paired *t*‐test

To check physical fitness, the schoolchildren took the test before (T0) and after the program period. We observed a significant improvement in all fitness skills (vertical jump, Figure 4; SBJ, Figure 5; rope jumps, Figure 6) in the group that participated in our intervention while no improvement was observed in the children of the control group (*p* < 0.0001 by paired Student's *t*‐test in all intervention groups; Figures 4, 5, 6).

## 4 DISCUSSION

Obesity, a syndrome with a multifactorial etiology, has been increasing continuously over previous decades most likely due to adverse changes of demographic and environmental factors (Afshin et al., 2017). Childhood with overweight and obesity is one of the most significant health problems (Abarca‐Gómez et al., 2017) with a high body mass index among children living in obesogenic household settings (Hüls et al., 2021), or whose parents have lower educational levels (Silventoinen et al., 2019) and, again, with a sedentary lifestyle (Hüls et al., 2021). The highest levels of children with overweight and obesity have been reported in Southern Europe (Spinelli et al., 2019) and especially for children growing up in low‐income families (Rossen & Schoendorf, 2012). Socio‐economic variability in childhood obesity and related lifestyles have also been reported in Italy (Lauria et al., 2015), where the prevalence is higher in southern regions where socio‐economic conditions are worst (Nardone et al., 2015). Since 2007, following the recommendations of the WHO European Ministerial Conference on Counteracting Obesity (WHO report, 2007), the Italian Minister of Health has promoted and funded the National Nutritional Surveillance System called “OKkio alla SALUTE” (Binkin et al., 2010; Spinelli et al., 2008), which enables comparisons to be made between Italian Regions (Lauria et al., 2019).

In the present paper we observed that out of the total students of both schools the number of overweight students was equal to 20.6% (23% of boys and 18% of girls), in accordance with what previously reported by others (Binkin et al., 2010; Lauria et al., 2019; Spinelli et al., 2008). As regards the prevalence rates of obesity among primary schoolchildren, it should be noted that in Italy these significantly decreased in the period from 2008 to 2019 (in Puglia region it reached 10%, International Obesity Task Force data). As this study was conducted from 2017 to 2018 these higher obesity rates (12%) are not caused by the pandemic‐dependent blockade of COVID‐19, but by preexisting socio‐economic conditions and other health indicators.

In the past few years, numerous interventions promoting healthy lifestyles, such as increasing PA and improving eating habits in the schools, have been implemented. Some of these studies have shown that school‐based interventions can be effective in preventing obesity and promoting PA (Van Barbeau et al., 2007; Kriemler et al., 2010; de Kop et al., 2019). On the other hand, there are also studies that do not report significant improvements in obesity, BMI, BP, and sedentary behavior following the various interventions (Van Barbeau et al., 2007; Kriemler et al., 2010; de Kop et al., 2019). Furthermore, some studies were performed without an adequate control group (van Sluijs et al., 2007).

In this context, and in the belief that school PA interventions are useful for preventing or treating obesity and overweight, also because low levels of PA have been associated with the risk of obesity (Owen et al., 2005), we examined the effect a 6‐month pilot intervention on students from two schools, located in two small towns with similar socio‐economic conditions. Nowadays, children spend their free time watching television and games in laptops, smartphones, and tablets. This has a noticeable metabolic rate lowering effect, due to a shift in PA (Owen et al., 2005) and increased calorie ingestion while watching or even caused by the effects of television advertising (Zenzen & Kridli, 2009).

From the interviews with the children, it emerges that 70.8% practice sports 2 or 3 times a week while 46% prefer to spend their free time watching TV, video games, and using other electronic media. As a result, 90% of children spend the afternoon sitting or lying down for more than two and a half hours. Physical activity guidelines prescribe a range of moderate to vigorous intensity activities for children and young adults for at least 60 min per day, but most fall well short of this recommendation. From this point of view, our data were worrying. Therefore, we have proposed a school‐based PA intervention as part of an obesity prevention or treatment program. The results we obtained show that our intervention program, despite the individual variability, induced a reduction in both the average value of the BMI and the waistline in the children of the intervention group; the latter result is of particular importance given the association between abdominal circumference in adults and cardiovascular morbidity. A previous study found that larger waist circumference correlates with higher concentrations of low‐density lipoprotein cholesterol, triglycerides, and insulin and lower concentrations of high‐density lipoprotein cholesterol in young people (Freedman et al., 1999; Kansra et al., 2021). Furthermore, waist circumference and the WHR index are believed to be specific markers of excessive fat accumulation in the upper body of children (McCarthy et al., 2001; Stodden et al., 2015). In fact, waist circumference appears to be more sensitive than BMI to identify children at risk of developing metabolic complications (McCarthy et al., 2001). In the present study 24 children (23%) had a WHR value >0.500 at T0; these children should be considered at high risk of developing cardiometabolic comorbidities. Fortunately, a positive influence of school‐based PA on the modulation of these adiposity markers was observed. Although the decrease in waist fat is slower and more difficult than in total fat, the decrease in waistline and WHR measured in the intervention group demonstrates that increased PA reduces the risk of developing cardiovascular comorbidities and acute coronary events later in life. As for sedentary behaviors, the experimental group responded positively to the program with the increase in the percentage of children who chose to walk or cycle. In this group there was also an increase in the afternoon hours dedicated to sport and at the same time a significant decrease in the hours spent using video games/PCs/smartphones/tablets, and TV.

The predictive utility of physical fitness tests in young people has not yet been fully demonstrated by the existing literature (i.e., musculoskeletal flexibility and endurance). We here examined the effects of the physical education program on physical fitness using three different tests. Improving physical fitness should be a goal of any PA intervention and, in fact, physical test results showed better physical fitness of the children in the experimental group. Several studies have shown a relationship between power tests requiring a single maximum effort at a submaximal speed (vertical and horizontal jump) and health indicators (such as body composition) in young people (Ingle et al., 2006; Minck et al., 2000; Van der Heijden et al., 2010). The standing long jump (used as a test of muscular strength, power and explosive strength of the lower body) is strongly correlated with the vertical jump (a lower‐ and upper‐extremity‐based power test) and is associated with musculoskeletal fitness in young people (Stodden et al., 2015). Rope jumping, an easily accessible mode of exercise, also has important implications for childhood obesity and the development of cardiovascular disease (Kim et al., 2020; Sung et al., 2019). Children in our experimental group, especially boys, greatly improved test performance, supporting the association between health‐related physical fitness (cardiorespiratory and musculoskeletal fitness, flexibility, and body composition) and health indicators in young people. Our intervention program for low‐income schools slowed the epidemic increase in the risk of overweight or obesity that was observed in the children in the control group.

In conclusion, since sedentary behaviors and a low level of PA negatively affect body weight across childhood, it is important to adopt a health‐related educational strategy, in order to promote an active and healthy lifestyle in children. The increase in PA has multiple health benefits in young people and it is therefore necessary to promote it throughout the day. The result of this study is expected to assist educational providers with physical activity program development. Thus, school administrators and teachers should be encouraged to design new health‐related physical education programs that change the lifestyle of school‐age children. Interactions between parental and school education, indicate that system level interventions might be better suited than individual intervention strategies.

## CONFLICT OF INTEREST

The authors declare that they have no competing interest and they have ethics approval and consent to participate.

## AUTHOR CONTRIBUTIONS

Antonella Muscella, Salvatore Colazzo, Santo Marsigliante and Antonio Di Maglie designed the study. Antonio Di Maglie collected and provided physical fitness and analyzed the data. Giulia My analyzed the data. Antonella Muscella generated the figures and wrote the paper. Santo Marsigliante did the Writing–review and the editing. All authors read and approved the final manuscript. The manuscript has not been published or submitted for publication elsewhere.

## References

[phy215115-bib-0001] Abarca‐Gómez, L. , Abdeen, Z. A. , Hamid, Z. A. , Abu‐Rmeileh, N. M. , Acosta‐Cazares, B. , Acuin, C. , Adams, R. J. , Aekplakorn, W. , Afsana, K. , Aguilar‐Salinas, C. A. , Agyemang, C. , Ahmadvand, A. , Ahrens, W. , Ajlouni, K. , Akhtaeva, N. , Al‐Hazzaa, H. M. , Al‐Othman, A. R. , Al‐Raddadi, R. , Al Buhairan, F. , … Ezzati, M. ; NCD Risk Factor Collaboration (NCD‐RisC) . (2017). Worldwide trends in body‐mass index, underweight, overweight, and obesity from 1975 to 2016: A pooled analysis of 2416 population‐based measurement studies in 128.9 million children, adolescents, and adults. Lancet, 390(10113), 2627–2642. 10.1016/S0140-6736(17)32129-3 29029897 PMC5735219

[phy215115-bib-0002] Afshin, A. , Forouzanfar, M. H. , Reitsma, M. B. , Sur, P. , Estep, K. , Lee, A. , Marczak, L. , Mokdad, A. H. , Moradi‐Lakeh, M. , Naghavi, M. , Salama, J. S. , Vos, T. , Abate, K. H. , Abbafati, C. , Ahmed, M. B. , Al‐Aly, Z. , Alkerwi, A. , Al‐Raddadi, R. , Amare, A. T. , … Murray, C. J. L. (2017). Health effects of overweight and obesity in 195 countries over 25 years. New England Journal of Medicine, 377(1), 13–27. 10.1056/NEJMoa1614362 PMC5477817 28604169

[phy215115-bib-0003] Barbeau, P. , Johnson, M. H. , Howe, C. A. , Allison, J. , Davis, C. L. , Gutin, B. , & Lemmon, C. R. (2007). Ten months of exercise improves general and visceral adiposity, bone, and fitness in black girls. Obesity, 15(8), 2077–2085. 10.1038/oby.2007.247 17712126

[phy215115-bib-0004] Battaglia, G. , Giustino, V. , Tabacchi, G. , Lanza, M. , Schena, F. , Biino, V. , Giuriato, M. , Gallotta, M. C. , Guidetti, L. , Baldari, C. , Gennaro, A. , Palma, A. , & Bellafiore, M. (2021). Interrelationship between age, gender, and weight status on motor coordination in Italian children and early adolescents aged 6–13 years old. Frontiers in Pediatrics, 9, 965. 10.3389/fped.2021.738294 PMC8461257 34568243

[phy215115-bib-0005] Binkin, N. , Fontana, G. , Lamberti, A. , Cattaneo, C. , Baglio, G. , Perra, A. , & Spinelli, A. (2010). A national survey of the prevalence of childhood overweight and obesity in Italy. Obesity Reviews, 11(1), 2–10. 10.1111/j.1467-789X.2009.00650.x 19761508

[phy215115-bib-0006] Buchheit, M. , Rabbani, A. , & Beigi, H. T. (2014). Predicting changes in high‐intensity intermittent running performance with acute responses to short jump rope workouts in children. Journal of Sports Science and Medicine, 13(3), 476–482. 25177172 PMC4126281

[phy215115-bib-0007] Bülbül, S. (2020). Exercise in the treatment of childhood obesity. Turkish Archives of Pediatrics, 55(1), 2–10. 32231444 10.14744/TurkPediatriArs.2019.60430 PMC7096559

[phy215115-bib-0009] Claver, F. , Jiménez, R. , Gil‐Arias, A. , Moreno, A. , & Moreno, M. P. (2017). The cognitive and motivation intervention program in youth female volleyball players. Journal of Human Kinetics, 59, 55–65. 10.1515/hukin-2017-0147 29134048 PMC5680686

[phy215115-bib-0010] Davies, B. N. (1990). The relationship of lean limb volume to performance in the handgrip and standing long jump tests in boys and girls, aged 11.6‐13.2 years. European Journal of Applied Physiology and Occupational Physiology, 60(2), 139–143. 10.1007/BF00846034 2335172

[phy215115-bib-0011] Dobbins, M. , De Corby, K. , Robeson, P. , Husson, H. , & Tirilis, D. (2009). School‐based physical activity programs for promoting physical activity and fitness in children and adolescents aged 6–18. Cochrane Database Systematic Review, 1: CD007651. 10.1002/14651858.CD007651 19160341

[phy215115-bib-0012] Freedman, D. S. , Serdula, M. K. , Srinivasan, S. R. , & Berenson, G. S. (1999). Relation of circumferences and skinfold thicknesses to lipid and insulin concentrations in children and adolescents: The Bogalusa Heart Study. American Journal of Clinical Nutrition, 69, 308–317. 10.1093/ajcn/69.2.308 9989697

[phy215115-bib-0013] Hall, C. J. S. , Eyre, E. L. J. , Oxford, S. W. , & Duncan, M. J. (2018). Relationships between motor competence, physical activity, and obesity in British Preschool Aged Children. Journal of Functional Morphology and Kinesiology, 3, 57. 10.3390/jfmk3040057 PMC7739285 33466985

[phy215115-bib-0014] Harris, K. C. , Kuramoto, L. K. , Schulzer, M. , & Retallack, J. E. (2009). Effect of school‐based physical activity interventions on body mass index in children: A meta‐analysis. CMAJ, 180, 719–726. 10.1503/cmaj.080966 19332753 PMC2659836

[phy215115-bib-0015] Hüls, A. , Wright, M. N. , Bogl, L. H. , Kaprio, J. , Lissner, L. , Molnár, D. , Moreno, L. A. , De Henauw, S. , Siani, A. , Veidebaum, T. , Ahrens, W. , Pigeot, I. , & Foraita, R. (2021). Polygenic risk for obesity and its interaction with lifestyle and sociodemographic factors in European children and adolescents. International Journal of Obesity, 45(6), 1321–1330. 10.1038/s41366-021-00795-5 33753884 PMC8159747

[phy215115-bib-0016] Hung, L.‐S. , Tidwell, D. K. , Hall, M. E. , Lee, M. L. , Briley, C. A. , & Hunt, B. P. (2015). A meta‐analysis of school‐based obesity prevention programs demonstrates limited efficacy of decreasing childhood obesity. Nutrition Research, 35, 229–240. 25656407 10.1016/j.nutres.2015.01.002

[phy215115-bib-0017] Hynynen, S. T. , van Stralen, M. M. , Sniehotta, F. F. , Araújo‐Soares, V. , Hardeman, W. , Chinapaw, M. J. M. , Vasankari, T. , & Hankonen, N. (2016). A systematic review of school‐based interventions targeting physical activity and sedentary behaviour among older adolescents. International Review of Sport and Exercise Psychology, 9, 22–44. 26807143 10.1080/1750984X.2015.1081706 PMC4706019

[phy215115-bib-0018] Ingle, L. , Sleap, M. , & Tolfrey, K. (2006). The effect of a complex training and detraining programme on selected strength and power variables in early pubertal boys. Journal of Sports Sciences, 24, 987–997. 10.1080/02640410500457117 16882633

[phy215115-bib-0019] Ip, P. , Ho, F.‐W. , Louie, L.‐T. , Chung, T.‐H. , Cheung, Y.‐F. , Lee, S.‐L. , Hui, S.‐C. , Ho, W.‐Y. , Ho, D.‐Y. , Wong, W.‐S. , & Jiang, F. (2017). Childhood obesity and physical activity‐friendly school environments. Journal of Pediatrics, 191, 110–116. 10.1016/j.jpeds.2017.08.017 28987751

[phy215115-bib-0020] Jiang, J. , Xia, X. , Greiner, T. , Wu, G. , Lian, G. , & Rosenqvist, U. (2007). The effects of a 3‐year obesity intervention in schoolchildren in Beijing. Child: Care, Health and Development, 33(5), 641–646. 10.1111/j.1365-2214.2007.00738.x 17725789

[phy215115-bib-0021] Kansra, A. R. , Lakkunarajah, S. , & Jay, M. S. (2021). Childhood and adolescent obesity: A review. Frontiers in Pediatrics, 8, 581461. 10.3389/fped.2020.581461 33511092 PMC7835259

[phy215115-bib-0022] Karuc, J. , & Mišigoj‐Duraković, M. (2019). Relation between weight status, physical activity, maturation, and functional movement in adolescence: An overview. Journal of Functional Morphology and Kinesiology, 4, 31. 10.3390/jfmk4020031 PMC7739286 33467346

[phy215115-bib-0023] Kim, J. , Son, W. M. , Headid Iii, R. J. , Pekas, E. J. , Noble, J. M. , & Park, S. Y. (2020). The effects of a 12‐week jump rope exercise program on body composition, insulin sensitivity, and academic self‐efficacy in obese adolescent girls. Journal of Pediatric Endocrinology and Metabolism, 33(1), 129–137. 10.1515/jpem-2019-0327 31812946

[phy215115-bib-0025] Kriemler, S. , Zahner, L. , Schindler, C. , Meyer, U. , Hartmann, T. , Hebestreit, H. , Brunner‐La Rocca, H. P. , van Mechelen, W. , & Puder, J. J. (2010). Effect of school based physical activity programme (KISS) on fitness and adiposity in primary schoolchildren: Cluster randomised controlled trial. BMJ, 340, c785. 10.1136/bmj.c785 20179126 PMC2827713

[phy215115-bib-0026] Kuczmarski, R. J. , Ogden, C. L. , Guo, S. S. , Grummer‐Strawn, L. M. , Flegal, K. M. , Mei, Z. , Wei, R. , Curtin, L. R. , Roche, A. F. , & Johnson, C. L. (2002). 2000 CDC growth charts for the United States: Methods and development. Vital and Health Statistics, 11(246):1–190. 12043359

[phy215115-bib-0027] Lauria, L. , Spinelli, A. , Buoncristiano, M. , & Nardone, P. (2019). Decline of childhood overweight and obesity in Italy from 2008 to 2016: Results from 5 rounds of the population‐based surveillance system. BMC Public Health, 19(1), 618. 10.1186/s12889-019-6946-3 31113403 PMC6528349

[phy215115-bib-0028] Lauria, L. , Spinelli, A. , Cairella, G. , Censi, L. , Nardone, P. , & Buoncristiano, M. ; 2012 Group OKkio alla SALUTE . (2015). Dietary habits among children aged 8‐9 years in Italy. Annali Dell'istituto Superiore Di Sanità, 51(4):371–381. 10.4415/ANN_15_04_20 26783227

[phy215115-bib-0029] Lavelle, H. V. , Mackay, D. F. , & Pell, J. P. (2012). Systematic review and meta‐analysis of school‐based interventions to reduce body mass index. Journal of Public Health, 34(3), 360–369. 10.1093/pubmed/fdr116 22267291

[phy215115-bib-0030] Li, X. , Lin, S. , Lin, S. , Guo, H. , Huang, Y. , Wu, L. , Zhang, Z. , Ma, J. , & Wang, H.‐J. (2014). Effectiveness of a school‐based physical activity intervention on obesity in school children: A nonrandomized controlled trial. BMC Public Health, 14(1), 10.1186/1471-2458-14-1282 PMC4320634 25510313

[phy215115-bib-0031] Liu, A. L. , Hu, X. Q. , Ma, G. S. , Cui, Z. H. , Pan, Y. P. , Chang, S. Y. , Zhao, W. H. , & Chen, C. M. (2007). Report on childhood obesity in China (6) evaluation of a classroom‐based physical activity promotion program. Biomedical and Environmental Sciences, 20(1), 19–23. 17458137

[phy215115-bib-0032] Lopes, V. P. , Stodden, D. F. , Bianchi, M. M. , Maia, J. A. , & Rodrigues, L. P. (2012). Correlation between BMI and motor coordination in children. Journal of Science and Medicine in Sport, 15(1), 38–43. 10.1016/j.jsams.2011.07.005 21831708

[phy215115-bib-0033] McCarthy, H. D. , & Ashwell, M. (2006). A study of central fatness using waist‐to‐height ratios in UK children and adolescents over two decades supports the simple message–'keep your waist circumference to less than half your height'. International Journal of Obesity, 30(6), 988–992. 10.1038/sj.ijo.0803226 16432546

[phy215115-bib-0034] McCarthy, H. D. , Jarrett, K. V. , & Crawley, H. F. (2001). The development of waist circumference percentiles in British children aged 5.0‐16.9 y. European Journal of Clinical Nutrition, 55(10), 902–907. 11593353 10.1038/sj.ejcn.1601240

[phy215115-bib-0036] Minck, M. R. , Ruiter, L. M. , Van Mechelen, W. , Kemper, H. C. G. , & Twisk, J. W. R. (2000). Physical fitness, body fatness, and physical activity: The Amsterdam Growth and Health Study. American Journal of Human Biology, 2, 593–599. 10.1002/1520-6300(200009/10)12:5<593:AID-AJHB3>3.0.CO;2-U 11534051

[phy215115-bib-0037] Musumeci, G. (2016). Physical activity for health ‐ an overview and an update of the physical activity guidelines of the italian ministry of health. Journal of Functional Morphology and Kinesiology, 1, 269–275. 10.3390/jfmk1030269

[phy215115-bib-0038] Nardone, P. , Spinelli, A. , Lauria, L. , Buoncristiano, M. , Bucciarelli, M. , & Galeone, D. ; Gruppo OKkio alla SALUTE . (2015). Variabilità sociodemografica nelle prevalenze di sovrappeso e obesità dei bambini in Italia nel 2014 [Sociodemographic variation in childhood overweight and obesity in Italy in 2014]. Epidemiologia E Prevenzione. 39(1):64. Italian. 25855552

[phy215115-bib-0039] Owen, C. G. , Martin, R. M. , Whincup, P. H. , Smith, G. D. , & Cook, D. G. (2005). Effect of infant feeding on the risk of obesity across the life course: A quantitative review of published evidence. Pediatrics, 115(5), 1367–1377. 10.1542/peds.2004-1176 15867049

[phy215115-bib-0040] Rodrigues, L. P. , Leitão, R. , & Lopes, V. P. (2013). Physical fitness predicts adiposity longitudinal changes over childhood and adolescence. Journal of Science and Medicine in Sport, 16(2), 118–123. 10.1016/j.jsams.2012.06.008 22824312

[phy215115-bib-0041] Rodrigues, L. P. , Stodden, D. F. , & Lopes, V. P. (2016). Developmental pathways of change in fitness and motor competence are related to overweight and obesity status at the end of primary school. Journal of Science and Medicine in Sport, 19(1), 87–92. 10.1016/j.jsams.2015.01.002 25660571

[phy215115-bib-0042] Rossen, L. M. , & Schoendorf, K. C. (2012). Measuring health disparities: Trends in racial‐ethnic and socioeconomic disparities in obesity among 2‐ to 18‐year old youth in the United States, 2001–2010. Annals of Epidemiology, 22(10), 698–704. 10.1016/j.annepidem.2012.07.005 22884768 PMC4669572

[phy215115-bib-0043] Sallis, J. F. , Prochaska, J. J. , & Taylor, W. C. (2000). A review of correlates2009 of physical activity of children and adolescents. Medicine and Science in Sports and Exercise, 32(5), 963–975. 10795788 10.1097/00005768-200005000-00014

[phy215115-bib-0044] Sargent, D. A. (1921). The physical test of a man. American Physical Education Review, 26, 188–194. 10.1080/23267224.1921.10650486

[phy215115-bib-0045] Silventoinen, K. , Jelenkovic, A. , Latvala, A. , Yokoyama, Y. , Sund, R. , Sugawara, M. , Tanaka, M. , Matsumoto, S. , Aaltonen, S. , Piirtola, M. , Freitas, D. L , Maia, J. A. , Öncel, S. Y. , Aliev, F. , Ji, F. , Ning, F. , Pang, Z. , Rebato, E. , Saudino, K. J. , … Kaprio, J. (2019). Parental education and genetics of BMI from infancy to old age: A pooled analysis of 29 twin cohorts. Obesity. 27(5):855–865. 30950584 10.1002/oby.22451 PMC6478550

[phy215115-bib-0046] Spinelli, A. , Baglio, G. , Cattaneo, C. , Fontana, G. , Lamberti, A. ; Gruppo OKkio alla SALUTE; Coorte PROFEA anno 2006 . (2008). OKkIO alla SALUTE: Promozione della salute e crescita sana nei bambini della scuola primaria [Promotion of healthy life style and growth in primary school children (OKkio alla SALUTE)]. Annale Di Igiene, 20(4):337–344. Italian. 19014105

[phy215115-bib-0047] Spinelli, A. , Buoncristiano, M. , Kovacs, V. A. , Yngve, A. , Spiroski, I. , Obreja, G. , Starc, G. , Pérez, N. , Rito, A. I. , Kunešová, M. , Sant’Angelo, V. F. , Meisfjord, J. , Bergh, I. H. , Kelleher, C. , Yardim, N. , Pudule, I. , Petrauskiene, A. , Duleva, V. , Sjöberg, A. , … Breda, J. (2019). Prevalence of severe obesity among primary school children in 21 European countries. Obesity Facts, 12(2), 244–258. 10.1159/000500436 31030201 PMC6547273

[phy215115-bib-0048] Stodden, D. , Sacko, R. , & Nesbitt, D. (2015). A review of the promotion of fitness measures and health outcomes in youth. American Journal of Lifestyle Medicine, 11(3), 232–242. 10.1177/1559827615619577 30202338 PMC6125085

[phy215115-bib-0049] Sung, K. D. , Pekas, E. J. , Scott, S. D. , Son, W. M. , & Park, S. Y. (2019). The effects of a 12‐week jump rope exercise program on abdominal adiposity, vasoactive substances, inflammation, and vascular function in adolescent girls with prehypertension. European Journal of Applied Physiology, 119(2), 577–585. 10.1007/s00421-018-4051-4 30554386

[phy215115-bib-0050] Tabacchi, G. , Bianco, A. , Alessi, N. , Filippi, A. R. , Napoli, G. , Jemni, M. , Censi, L. , Breda, J. , Schumann, N. L. , Firenze, A. , Vitale, F. , & Mammina, C. (2016). Design, implementation, and evaluation of the adolescents and surveillance system for the obesity prevention project. Medicine, 95(12), e3143. 10.1097/MD.0000000000003143 27015195 PMC4998390

[phy215115-bib-0051] Van de Kop, J. H. , van Kernebeek, W. G. , Otten, R. H. J. , Toussaint, H. M. , & Verhoeff, A. P. (2019). School‐based physical activity interventions in prevocational adolescents: A systematic review and meta‐analyses. Journal of Adolescent Health, 65, 185–194. 10.1016/j.jadohealth.2019.02.022 31202623

[phy215115-bib-0052] Van der heijden, G.‐J. , Wang, Z. J. , Chu, Z. , Toffolo, G. , Manesso, E. , Sauer, P. J. J. , & Sunehag, A. L. (2010). Strength exercise improves muscle mass and hepatic insulin sensitivity in obese youth. Medicine and Science in Sports and Exercise, 42(11), 1973–1980. 10.1249/MSS.0b013e3181df16d9 20351587 PMC2944907

[phy215115-bib-0053] van Sluijs, E. M. , McMinn, A. M. , & Griffin, S. J. (2007). Effectiveness of interventions to promote physical activity in children and adolescents: Systematic review of controlled trials. BMJ, 335(7622), 703. 10.1136/bmj.39320.843947.BE 17884863 PMC2001088

[phy215115-bib-0054] von Hippel, P. T. , Powell, B. , Downey, D. B. , & Rowland, N. J. (2007). The effect of school on overweight in childhood: Gain in body mass index during the school year and during summer vacation. American Journal of Public Health, 97(4), 696–702. 10.2105/AJPH.2005.080754 17329660 PMC1829359

[phy215115-bib-0055] Waters, E. , de Silva‐Sanigorski, A. , Hall, B. J. , Brown, T. , Campbell, K. J. , Gao, Y. , Armstrong, R. , Prosser, L. , & Summerbell, C. D. (2011). Interventions for preventing obesity in children. Cochrane Database Systematic Review, 12, CD001871‐kri. 10.1002/14651858.CD001871.pub3 22161367

[phy215115-bib-0056] WHO European Ministerial Conference on Counteracting Obesity . (2007) Report of a WHO conference. World Health Organization; 1–30.

[phy215115-bib-0057] Yuksel, H. S. , Şahin, F. N. , Maksimovic, N. , Drid, P. , & Bianco, A. (2020). School‐based intervention programs for preventing obesity and promoting physical activity and fitness: A systematic review. International Journal of Environmental Research and Public Health, 17(1), 347. 10.3390/ijerph17010347 PMC6981629 31947891

[phy215115-bib-0058] Zenzen, W. , & Kridli, S. (2009). Integrative review of school‐based childhood obesity prevention programs. Journal of Pediatric Health Care, 23(4), 242–258. 10.1016/j.pedhc.2008.04.008 19559992

[phy215115-bib-0059] Zhu, Y. , Shao, Z. , Jing, J. , Ma, J. , Chen, Y. , Li, X. , Yang, W. , Guo, L. I. , & Jin, Y. U. (2016). Body mass index is better than other anthropometric indices for identifying dyslipidemia in Chinese children with obesity. PLoS One, 11(3), e0149392. 10.1371/journal.pone.0149392 26963377 PMC4786269

